# Separation and Quantification of Four Main Chiral Glucosinolates in *Radix Isatidis* and Its Granules Using High-Performance Liquid Chromatography/Diode Array Detector Coupled with Circular Dichroism Detection

**DOI:** 10.3390/molecules23061305

**Published:** 2018-05-29

**Authors:** Yanhong Shi, Cheng Zheng, Jinhang Li, Li Yang, Zhengtao Wang, Rui Wang

**Affiliations:** 1 The MOE Key Laboratory for Standardization of Chinese Medicines, Institute of Chinese Materia Medica, Shanghai University of Traditional Chinese Medicine, Shanghai 201203, China; shiyhtcm@shutcm.edu.cn (Y.S.); yl7@shutcm.edu.cn (L.Y.); wangzht@hotmail.com (Z.W.); 2 The SATCM Key Laboratory for New Resources and Quality Evaluation of Chinese Medicines, Institute of Chinese Materia Medica, Shanghai University of Traditional Chinese Medicine, Shanghai 201203, China; 3 School of Pharmacy, Shanghai University of Traditional Chinese Medicine, Shanghai 201203, China; zhengcheng4231@yeah.net (C.Z.); leejh310@163.com (J.L.); 4 Center for Chinese Medical Therapy and Systems Biology, Shanghai University of Traditional Chinese Medicine, Shanghai 201203, China

**Keywords:** *Radix Isatidis*, glucosinolates, HPLC-UV-CD, chiral analysis, quantification

## Abstract

As chemical drugs, separation and quantification of the specific enantiomer from the chiral compounds in herbal medicines are becoming more important. To clarify the chemical characterization of chiral glucosinolates—the antiviral active ingredients of *Radix Isatidis*, an optimized efficient method of HPLC-UV-CD was developed to simultaneously separate and quantify the four main chiral glucosinolates: progoitrin, epiprogoitrin, and *R,S*-goitrin. The first step was to determine progoitrin, epiprogoitrin, and *R,S*-goitrin using HPLC-UV, and then determine the *R*-goitrin and *S*-goitrin by coupling with CD detection. Subsequently, through the linear relations between anisotropy factor (*g* factor) and the percent optical purity of *R*-goitrin, the contents of *R*-goitrin and *S*-goitrin from the *R,S*-goitrin mixture were calculated separately. Furthermore, the chemical composition features of the four chiral glucosinolates in 37 samples from crude drugs, decoction pieces, and granules of *R. Isatidis* were conducted. The total content of the four glucosinolates was obviously higher in crude drugs, and the variance character of each glucosinolate contents was different. In summary, the accurate measurement method reported here allows for better control of the internal quality of *R. Isatidis* and its granules and provides a powerful approach for the analysis of other chiral components in traditional Chinese medicines.

## 1. Introduction

Natural products from traditional Chinese medicines (TCMs) represent a major part of today’s pharmaceutical market as they possess a number of biological activities, such as antimicrobial, antitumor, anti-inflammatory, antiviral, cardiovascular activities, and so on [1]. Since chirality is a fundamental characteristic of nature, a large number of well-known therapeutic ingredients from natural products of TCMs are chiral. Here, one isomer possesses a desired therapeutic effect, whereas its paired enantiomer could be inactive or even have undesirable effects [2]. For example, *S*-ibuprofen, the first chiral drugs of the non-steroidal anti-inflammatory drug approved in 1994, was over a 100-fold more potent as an inhibitor of cyclooxygenase 1 enzyme than *R*-ibuprofen [3]. Notably, 45 new drugs approved by the US food and Drug Administration in 2015 were single enantiomers, except for lesinurad [4]. For this reason, investigating the biological activities of specific enantiomers of chiral compounds in TCMs is becoming more important. Chiral analysis of the chemical constituents in TCMs is therefore urgent and desired.

*Radix Isatidis*, the dried roots of *Isatis indigotica* Fort. (Fam. Brassicaceae), have been used to remove heat and toxins, cool the blood, and clear the throat in oriental countries for thousands of years [5,6,7]. Since 1985, it has been recorded in the Chinese Pharmacopoeia and recognized as an important traditional Chinese herb for the prevention and treatment of cold and malignant infectious diseases, especially SARS coronavirus and H1N1 influenza virus [8,9,10]. *R. Isatidis* and its granules have been occupying a huge share of the international market in recent decades.

The aqueous extract of *R. Isatidis* and their chiral glucosinolates, such as epiprogoitrin, progoitrin, and *R*,*S*-goitrin, have attracted more attention due to their antiviral activity [11,12,13]. In our previous studies, the specific marker of *R*,*S*-goitrin has been used for quality evaluation of *R. Isatidis* and its granules, which has been recorded in the Chinese Pharmacopoeia (2015 Edition) [6,14,15]. *R*-goitrin and *S*-goitrin were separately transferred from epiprogoitrin and progoitrin via the effect of myrosinase and considered to be the most noteworthy glucosinolate-biotransformed products [16]. The contents of degradation products might be influenced by the procedures of traditional processing and extraction in crude drugs, decoction pieces, and granules of *R. Isatidis* [17]. Furthermore, the pharmacological properties of *R. Isatidis* is related to *R*-goitrin. The inhibitory effect of *R*-goitrin against influenza viral neuraminidase was revealed by fluorescent enzyme immunoassay method [11,13], while *S*-goitrin is known as an antithyroid factor [18].

To date, X-ray crystallography, NMR, vibrational circular dichroism (VCD) and enantioselective chromatography have been frequently adopted to determine the chiral components of TCMs [1,2]. Several enantioselective chromatography such as reversed-phase high-performance liquid chromatography (RP-HPLC) coupled with the type of Chrialpak® IC chiral column [19,20] and supercritical fluid chromatography (SFC) have been used for chiral separation of *R*,*S*-goitrin in *R. Isatidis* [21,22]. However, these methods were limited owing to the complex operation, high cost, and lack of simultaneous quantification of other chiral glucosinolates. Therefore, it is desired and urgent to establish a more efficient enantioselective method to simultaneously quantify the four glucosinolates, and furthermore resolve *R*- and *S*-goitrin for the effective use and quality control of *R. Isatidis* and its granules.

In the present study, a high-performance liquid chromatography/diode array detector coupled with circular dichroism detection (HPLC-DAD-CD) was adopted to develop a more efficient method for simultaneous separation and quantification of the four chiral glucosinolates—epiprogoitrin, progoitrin, *R*-goitrin, and *S*-goitrin—in *R. Isatidis*. Progoitrin, epiprogoitrin and *R-* and *S*-goitrin were simultaneously determined using HPLC-UV, whereas *R*-goitrin was detected and quantified by coupling with CD detection. Through the linear relations between anisotropy factor (*g* = ΔA_CD_/A_UV_) and the percent optical purity of *R*-goitrin, the contents of *R*-goitrin and *S*-goitrin in the mixture *R,S-*goitrin was calculated. Furthermore, glucosinolate profiling of 37 samples from crude drugs, decoction pieces, and granules of *R. Isatidis* was carried out to clarify their chemical characteristics and to find their content distribution.

## 2. Results and Discussion

### 2.1. Optimization of HPLC-UV-CD Conditions

In order to obtain the best chemical information and separation mechanism in chromatograms, different HPLC parameters including various mobile gases, detection wavelengths, and gradient elution were compared. Comparison with different compositions of acetonitrile/methanol–water, methanol–water with different modifiers including formic acid and ammonium acetate showed the combination of methanol and 30 mM ammonium acetate (adjusted pH for 5.0 with formic acid) gave an optimal mobile phase system for glucosinolates separation. As shown in Figure S1, in order to acquire better peak intensity, five UV wavelengths—230, 242, 245, 254, 280 nm—were detected and 230 nm gave the best results. In addition, the maximum absorption CD wavelength of *R*- and *S*-goitrin was focused on 195 and 280 nm, respectively, whereas the detection range of the present CD-2095 detector was 220–490 nm. Therefore, the CD wavelength was set at 280 nm.

### 2.2. Method Validation of Quantitative Analysis

As shown in Table 1, all calibration curves of the glucosinolates showed good linearity (*R*^2^ > 0.9991) within a relatively wide range of concentration (0.0365–3.080 mg/mL). The limit of detection (LOD) and the limit of quantification (LOQ) were lower than 0.802 and 2.305 μg/mL, respectively.

The known purities of *R*-goitrin (%) and *S*-goitrin (%) were mixed and simultaneously determined by UV and CD to calculate the *g* factors (Table 2). The relative purities of *R*-goitrin (%) and *g* factors made calibration curves. The calibration curve between *g* factors (*X*_2_) and the different relative purities of *R*-goitrin (*Y*_2_) was *Y*_2_ = −0.0092*X*_2_ + 0.4965 (*R*^2^ = 0.9970), which showed a good linearity with a wide range of *R*-goitrin purities (0~100 %).

The precision, repeatability, and stability of the developed method were also validated for each analyte, and the RSD values were less than 3.0%. The recoveries of five glucosinolates were measured from 99.1 to 103.3%, and the RSD values were less than 3.0% (Table 3).

### 2.3. Glucosinolates Profiles of Radix Isatidis and Its Granules

**Figure 1:**
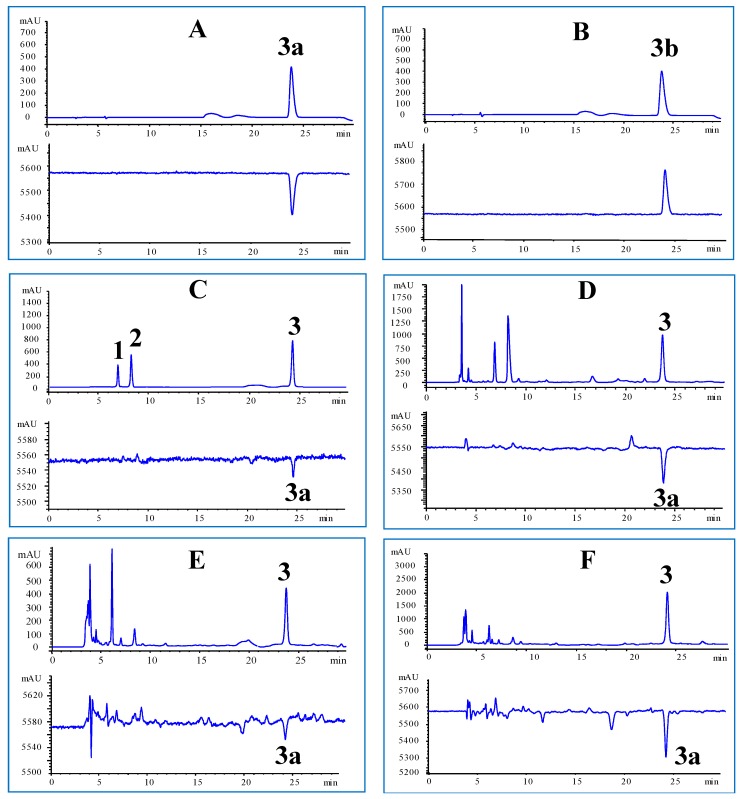
Representative UV (**upper**) and CD (**lower**) chromatograms of glucosinolates, crude drug, decoction pieces and granules of *Radix Isatidis*. (**A**) *R*-goitron, (**B**) *S*-goitrin, (**C**) mixed glucosinolate references, (**D**) crude drugs of *R. Isatidis* (B10), (**E**) decoction pieces of *R. Isatidis* (B22), (**F**) granules of *R. Isatidis* (B35). **1**: progoitrin, **2**: epiprogoitrin, **3**: *R,S*-goitrin, **3a**: *R*-goitrin, **3b**: *S*-goitrin.

Compared to previous published methods [19,20,21,22], glucosinolates profiles of crude drug, decoction pieces, and granules of *R. Isatidis* were performed using HPLC-UV-CD, which improved the limitation of high-cost chiral columns, complex operation of sample preparation, and simultaneous quantification of the characteristic glucosinolates. In the typical UV and CD chromatograms of *R. Isatidis* samples (Figure 1), *R-*goitrin and *S-*goitrin showed the same retention time, UV absorption feature, and overlapping peak. They could not be separated by UV detection without using the specific chiral columns (Figure 1A,B; upper). In contrast to that, *R*-goitrin and *S*-goitrin had completely opposite chromatographic profiles at the same retention time as shown in CD chromatograms (Figure 1A,B; lower). Notably, in the typical CD chromatograms, only *R*-goitrin could be detected in the mixed *R,S*-goitrin, crude drugs, decoction pieces, and granules of *R. Isatidis*. Therefore, the *R*-goitrin content could be quantified using CD detector, while the *S*-goitrin content was calculated using the mixed *R,S*-goitrin and the determined *R*-goitrin content. Combining the CD chromatographic properties with total content of the mixed *R,S*-goitrin, it provided an efficient method to determine the content of *R*-goitrin in the mixed *R,S*-goitrin without chiral separation.

As shown in Figure 1, the chemical composition of progoitrin, epiprogoitrin, and *R,S*-goitrin in crude drugs, decoction pieces, and granules of *R. Isatidis* showed that the chromatographic patterns were found to be consistent between all 37 samples but their contents obviously varied. The three analytes could be easily detected in the UV chromatograms, while only *R*-goitrin was determined in CD chromatograms. We think the different absorption intensity between UV and CD detection might be due to the differences in chemical structures. Therefore, the content variations of these characteristic glucosinolates should be conducted.

### 2.4. Chemical Comparison on the Basis of Contents of Four Glucosinolates

**Figure 2:**
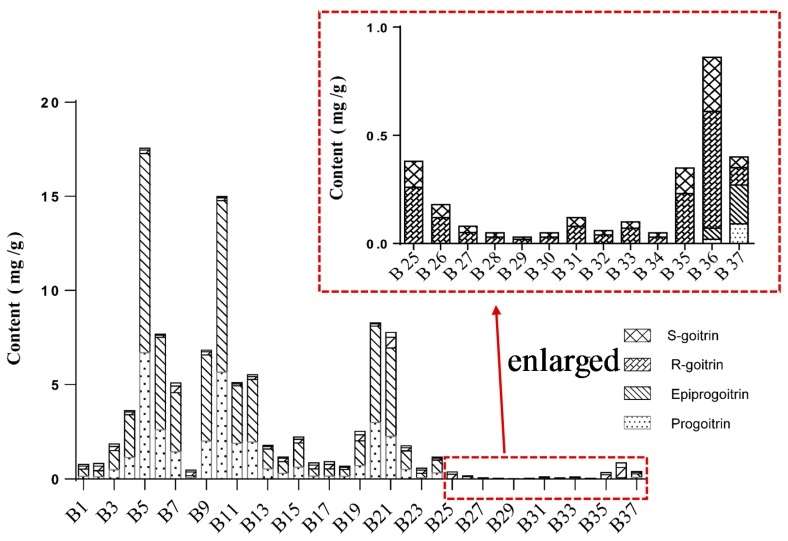
Contents of four glucosinolates in different crude drugs, decoction pieces, and granules of *Radix Isatidis*.

The contents of four glucosinolates—progoitrin, epiprogoitrin, *R*-goitrin, and *S*-goitrin—were simultaneously quantified in 37 samples using HPLC-UV coupled with CD detection. As shown in Figure 2 and Table S2, the total contents of four glucosinolates in three types of *R. Isatidis* were significantly different. It was obviously higher in crude drugs (0.77–17.54 mg/g, Mean ± SD: 5.04 ± 5.03) and decoction pieces (0.56–8.29 mg/g, Mean ± SD: 2.72 ± 3.07), while the glucosinolates occupied relatively lower contents in its granules (0.03–0.87 mg/g, Mean ± SD: 0.21 ± 0.24).

Most previous studies have focused only on the content of *R,S*-goitrin mixture in *R. Isatidis* and its related products. In the present study, *R*-goitrin accounted for a majority of the *R,S-*goitrin component in crude drugs, decoction pieces, and granules of *R. Isatidis*; the *R*-goitrin content was twice as much as that of *S*-goitrin. The results might provide positive evidence that *R*-goitrin is responsible for the pharmacological properties of *R. Isatidis*.

It was noticed that progoitrin and epiprogoitrin contents showed an obvious declining trend in crude drugs and decoction pieces of *R. Isatidis*. In particular, they could not be detected in most of the granule samples. Compared with crude drugs, *R*-goitrin and *S*-goitrin contents in decoction pieces increased slightly. The traditional processing and extraction methods could improve the biotransformation of progoitrin and epiprogoitrin, and then increase the content of the degradation products (*R*- and *S*-goitrin). This is consistent with our previous report [16,17]. However, the content dynamic change features of the glucosinolates in crude drugs, decoction pieces, and granules as well as the main influencing parameters for biotransformation among these glucosinolates need to be further investigated. 

## 3. Materials and Methods

### 3.1. Sample Materials

Fifteen crude drugs (B1–B15), nine decoction pieces (B16–B24), and thirteen granules (B25–B37) of *R. Isatidis* were collected from the main commercial herbal markets and different herbal manufactories in China (Table S1). The vouchers have been deposited in Shanghai R&D Centre for standardization of Chinese Medicines, Shanghai University of Traditional Chinese Medicine.

### 3.2. Chemicals and Reagents

**Figure 3:**
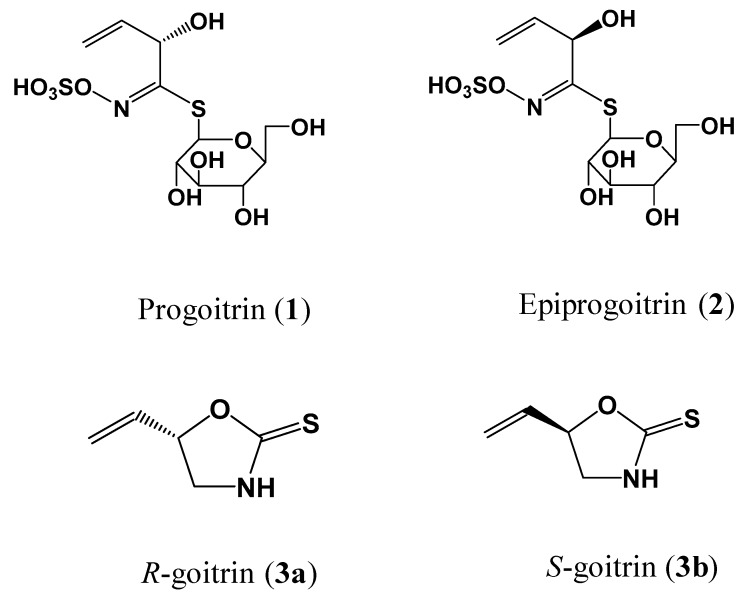
Chemical structures of four characteristic glucosinolates isolated from *Radix Isatidis.*

The four glucosinolates—epiprogoitrin, progoitrin, and *R*,*S*-goitrin—were isolated and purified from the root of *I. indigotica* Fort. in our laboratory. *R*-goitrin and *S*-goitrin were prepared from *R*,*S*-goitrin using the Shiseido^®^ CD-pH chiral column (5 μm, 250 × 4.6 mm) with acetonitrile–water (30:70, *v*/*v*). Their chemical structures (Figure 3) were elucidated by a series of spectroscopic and chemical analyses [23]. The purities of five glucosinolates were determined to be higher than 98.0% through HPLC-DAD analysis. HPLC-grade acetonitrile, methanol, formic acid (≥98.0%, HPLC grade), and ammonium acetate (≥98.0%, HPLC grade) for HPLC analysis were purchased from Thermo Fisher Scientific (Swedesboro, NJ, USA). Water was prepared by a Milli-Q^®^ system (Millipore, MA, USA). All other reagents of analytical grade for extraction were purchased from Tianjin Damao Chemical Reagent Factory (Tianjin, China).

### 3.3. Instrumentation and Chromatographic Conditions

HPLC system (Agilent 1260, Santa Clara, CA, USA) was equipped with a G1311 DAD, a quaternary pump and an online degasser. A CD detector (CD-2095, Jasco, Tokyo, Japan) was coupled with the DAD detector for the chiral analysis. The mobile phase consisted of methanol (A) and 30 mM ammonium acetate (adjusted pH for 5.0 with formic acid B). The sample solutions were analyzed on a Ultimate AQ-C_18_ column (5 μm, 250 × 4.6 mm) with a gradient elution system as follows: 0–10 min, 0% A; 10–15 min, 0–5% A; 15–25 min, 5% A. The flow rate was 1.0 mL/min, and the column temperature was set at 30 °C. UV and CD detective wavelengths were set at 230 nm and 280 nm, respectively.

### 3.4. Preparation of Standard Solutions

Each of the five reference compounds was accurately weighed and then dissolved in water to prepare the standard solutions of 2.084 mg/mL for progoitrin, 3.080 mg/mL for epiprogoitrin, 1.168 mg/mL for *R*,*S*-goitrin, 0.5488 mg/mL for *R*-goitrin, and 0.5080 mg/mL for *S*-goitrin. For UV detection, a series of standard solutions were prepared by appropriate dilution of the stock solutions to make calibration curves. The calibration curve was obtained by plotting peak area (*Y_1_*) of each reference glucosinolate against the concentration of the corresponding compound (*X_1_*). Furthermore, the known purities of *R*-goitrin (%) were determined by UV and CD to calculate the anisotropy factor (*g* = ΔA/A). The calibration curve depended on *g* factors (*Y_2_*) of each reference *R*-goitrin against the relative purities of *R*-goitrin (*X_2_*).

### 3.5. Preparation of Sample Solutions

***Crude drugs and decoction pieces***: The respective sample was pulverized to obtain homogeneous fine powder. 1.0 g of the fine powder was accurately weighed and extracted with 30 mL water by refluxing in a water bath for 1 h, then cooled and filtered. 1 mL of the filtrate was dissolved with 1 mL water containing 2% formic acid (*v*/*v*). The mixed solution was packed into a SPE column (Waters Oasis WAX 3cc Cartridge/ 60 mg, Waters, USA), which was activated with 3 mL methanol and then washed with 3 mL water before adding sample solution. The SPE column was orderly eluted with 3 mL water containing 2% formic acid, 2 mL methanol (A) and 2 mL methanol containing 5% ammonium hydroxide (*v*/*v*, B). Subsequently, the eluted solutions of A and B was dried by flushing with nitrogen (N_2_). The residue was dissolved with 0.1 mL methanol for HPLC analysis.

***Granules***: The granules were pulverized and screened through a 300 μm sieve to obtain homogeneous fine powder. 2.0 g (containing sugar) and 1.0 g (no sugar) of the fine powder was accurately weighed and extracted with 10 mL water by ultrasonication at room temperature for 15 min, then cooled and filtered. 1 mL of the filtrate was dissolved with 1mL water containing 2% formic acid (*v*/*v*). The mixed solution was packed into a SPE column. Subsequently, the sample solution was prepared through the same operation procedure with sample solutions of crude drugs and decoction pieces.

### 3.6. Method Validation

Linearity was assessed by generating six-point calibration curves for each reference compound. The precision was evaluated by replicate injections of a mixture solution containing four glucosinolates and a sample solution of *R. Isatidis* (B10). Six injections of the same preparation solutions per day were investigated for three days. Six preparation solutions of a sample (B10) were analyzed for repeatability. To determine the recovery rate of extraction, the four reference components (approximately 50%, 100% and 150% of the original amount) were added to the weighed powder of *R. Isatidis* that was extracted and analyzed.

### 3.7. Statistical Analysis

As the CD signal depends only on the enantiomeric composition of the chiral molecule whereas UV absorbance is related to the analyte concentration, the measurement method of the enantiomeric content from the chiral sample was referred to the reported literatures [24,25]. Firstly, the CD detector recorded both dichroic (ΔƐ or ΔA) and UV (Ɛ or A) signals at the optimized wavelength and calculated the anisotropy factor (*g* = ΔƐ/Ɛ or ΔA/A), which is dependent of the enantiomeric purity. Secondly, through the linear relations between *g* factor and the percent optical purity of the enantiomeric, the relative proportion of the enantiomeric in the chiral molecule were calculated. Finally, the absolute content of enantiomeric in the chiral molecule was measured by its relative purity and UV absorbance.

## 4. Conclusions

The present study proposed an efficient method of HPLC-UV-CD for simultaneous separation and quantification of the main chiral glucosinolates in *R. Isatidis*. Progoitrin, epiprogoitrin and *R,S*-goitrin were easily determined by UV detection, whereas *R*-goitrin and *S*-goitrin were separated by coupling with CD detection. Through the linear relations between *g* factor and the percent optical purities of *R*-goitrin, the contents of *R*-goitrin and *S*-goitrin were successfully calculated. Subsequently, glucosinolate profiles in crude drugs, decoction pieces and granules of *R. Isatidis* were conducted and found that the variance character of each of the glucosinolate contents was different. In summary, the new method allows better control of the internal quality of *R. Isatidis* and its granules and provides a powerful approach and foundation for further investigation of dynamic change features of the contents and the main influencing parameters for biotransformation of glucosinolates in *R. Isatidis*.

## Figures and Tables

**Table 1:** Regression equations, linear range, limit of detection (LOD) and limit of quantification (LOQ) for three analytes.

Glucosinolates	Calibration Curves Equation	*R* ^2^	Linear Range (mg/mL)	LOD (μg/mL)	LOQ (μg/mL)
Progoitrin	*Y* = 5.10 × 10^3^*X* − 22.43	1.0000	0.0651~2.084	0.583	1.885
Epiprogoitrin	*Y* = 6.27 × 10^3^*X* + 23.37	1.0000	0.0963~3.080	0.802	2.305
*R,S*-goitrin	*Y* = 3.51 × 10^4^*X* + 576.80	0.9991	0.0365~1.168	0.025	0.067

**Table 2:** The results of g factors from the different relative purities of *R*-goitrin using UV and CD detection.

*R-*goitrin	CD	UV	*g* Factor
(%)	∆A	A	∆A/A
100	−4391.9	10,317.8	−0.4257
80	−2414.5	10,293.2	−0.2346
65	−1026.8	10,113.3	−0.1015
50	281.2	10,092.9	0.0279
35	1715.0	10,078.7	0.1702
20	3118.2	10,114.5	0.3082
0	5024.0	9969.0	0.5040

**Table 3:** The results of precision, repeatability, stability, and recovery for five analytes.

Glucosinolates	Precision (RSD %)		Repeatability (RSD %) *n* = 6	Stability (RSD %) *n* = 6	Recovery	
	Intra-Day *n* = 6	Inter-Day *n* = 3			(Mean %) *n* = 9	RSD (%)
Progoitrin	1.23	0.15	1.56	0.37	99.1	2.53
Epiprogoitrin	1.98	0.16	1.41	0.05	99.3	2.58
*R,S*-goitrin	1.24	0.53	1.21	0.82	103.3	2.89
*R-*goitrin	1.35	0.96	1.28	0.64	101.5	2.95
*S*-goitrin	1.05	0.58	2.82	1.42	99.1	2.53

## Supplementary Materials

The following are available online. Figure S1: The CD absorbance features of *R*- and *S*-goitrin (A), maximum UV absorbance spectrum of the four glucosinolates (B), and the chemical profiling of the four glucosinolates in five different wavelength (C); Table S1: Crude drugs, decoction pieces and granules of *Radix Isatidis* used in this study; Table S2: The contents of four glucosinolates in 37 samples from crude drugs, decoction pieces and granules of *Radix Isatidis* (*n* = 3).
