# Supplementary material for: Separation and Quantification of Four Main Chiral Glucosinolates in Radix Isatidis and Its Granules Using High-Performance Liquid Chromatography/Diode Array Detector Coupled with Circular Dichroism Detection
Source: Molecules. 2018 May 29;23(6):1305. doi: 10.3390/molecules23061305 (PMC6100438; doi:10.3390/molecules23061305)
Supplement: Supplementary file 1 [file molecules-23-01305-s001.docx]

**Supporting information**

Separation and Quantification of Four Main Chiral Glucosinolates in *Radix Isatidis* and Its Granules Using High-Performance Liquid Chromatography/Diode Array Detector Coupled with Circular Dichroism Detection

Yanhong Shi, Cheng Zheng, Jinhang Li, Li Yang, Zhengtao Wang, Rui Wang

**Contents:**

**Figure S1** The CD absorbance features of *R*- and *S*-goitrin (A), maximum UV absorbance spectrum of the four glucosinolates (B), and the chemical profiling of the three glucosinolates in five different wavelength (C).

**Table S1** Crude drugs, decoction pieces and granules of *Radix Isatidis* used in this study.

**Table S2** The contents of four glucosinolates in 37 samples from crude drugs, decoction pieces and granules of *Radix Isatidis* (n=3).

**Figure S1** The CD absorbance features of *R*- and *S*-goitrin (A), maximum UV absorbance spectrum of the four glucosinolates (B), and the chemical profiling of the three glucosinolates in five different wavelength (C), **1**: progoitrin; **2**: epiprogoitrin; **3**: *R,S*-goitrin.

**Table S1** Crude drugs, decoction pieces and granules of *Radix Isatidis* used in this study.

**Table S2** The contents of four glucosinolates in 37 samples from crude drugs, decoction pieces and granules of *Radix Isatidis* (n=3).
